# Comparison of risk patterns in carcinoma and melanoma of the skin in men: a multi-centre case–case–control study

**DOI:** 10.1038/sj.bjc.6602982

**Published:** 2006-02-21

**Authors:** R Zanetti, S Rosso, C Martinez, A Nieto, A Miranda, M Mercier, D I Loria, A Østerlind, R Greinert, C Navarro, G Fabbrocini, C Barbera, H Sancho-Garnier, L Gafà, A Chiarugi, R Mossotti

**Affiliations:** 1 CPO – Registro Tumori Piemonte, via San Francesco da Paola 31, 10123 Turin, Italy; 2 Escuela Andaluza de Salud Pública, Registro de Cáncer de Granada, Campus de Cartuja, Apdo. Correos 2070, 18080 Granada, España; 3 Departamento de Ciencias Sociosanitarias, Facultad de Medicina Universidad de Sevilla, Avda. Sanchez Pizjuán s/n 41009, Sevilla, España; 4 Registo Oncológico Regional Sul, Instituto Portugûes de Oncologia, Rua Prof. Lima Basto, 1093 Lisboa, Portugal; 5 Registre des Tumeurs du Doubs, Faculté de Médecine-Pharmacie, Place Saint Jacques, 25030 Besançon Cédex, France; 6 Instituto de Oncología ‘A.H. Roffo’, Av. San Martín 5481, 1417 Buenos Aires, Argentina; 7 Dermatology Clinic, Slotsgade 14 A, 3400 Hillerød, Denmark; 8 Krankenhaus Buxtehude, Dermatologisches Zentrum, Am Krankenhaus 1, Buxtehude, Germany; 9 Consejería de Sanidad y Consumo, Servicio de Epidemiología, Ronda de Levante 11, 30008 Murcia, España; 10 Clinica Dermatologica Università ‘Federico II’ Azienda Universitaria Policlinico, via S. Pansini 5, Edificio 10, Napoli, Italy; 11 Ospedale degli Infermi di Biella, via Caraccio, Dermatologia, Biella, Italy; 12 Epidaure, Rue des Apothicaires, Parc Euromédecine, 34298 Montpellier, France; 13 Lega Italiana per la lotta contro i tumori Sezione provinciale Ragusa, via Traspontino 1, 97100 Ragusa, Italy; 14 Dipartimento di Dermatologia, Università di Firenze, via della Pergola 58/60, 10121 Firenze, Italy; 15 Istituto per lo Studio delle Macromolecole (ISMAC), Consiglio Nazionale delle Ricerche (CNR), Corso G. Pella 16, 13900 Biella, Italy

**Keywords:** skin cancer, melanoma, basal-cell carcinoma, squamous-cell carcinoma, case–case–control, sun exposure

## Abstract

We directly compared risk factors between 214 histologically confirmed melanomas (CMM), 215 basal-cell carcinomas (BCC) and 139 squamous-cell carcinomas (SCC) in a multiple case–case–control study with 349 controls from patients without dermatological disease admitted to the same hospitals. Subjects with fair hair had a significant risk increase for all types of tumours at a comparable level (OR_adj_ for blonde hair: CMM 2.3; SCC 2.4; BCC 2.3). The effect of pale eyes was significant and similar for CMM and BCC (OR_adj_ 2.6). Intermittent sun exposure measured in hours spent at beach during holidays was significant for both CMM (OR_adj_ 2.6 for more than 7000 lifelong hours) and BCC (OR_adj_ 2.1 for more than 7000 lifelong hours), while SCC exhibited a significant risk increase for chronic exposure to sunlight measured in hours of outdoor work (OR_adj_ 2.2 for more than 6000 lifelong hours). In the case–case comparison using a multinomial logistic regression model, we found a statistically significant risk difference for pale eyes, and number of naevi in the CMM group, compared to other skin cancers. For intermittent sun exposure, there was a significant risk difference of BCC when compared to the risk of SCC. Factors influencing risk of SCC are different, with chronic exposure to sun playing a major role in causing this type of carcinoma.

There has been extensive study of the complex interaction between different phenotypes and sun exposure in the genesis of CMM in different Caucasian populations across America (Lew *et al*, 1983; Elwood *et al*, 1985; Holly *et al*, 1987; Loria and Matos, 2001), Europe (MacKie and Aitchison, 1982; Østerlind *et al*, 1988; Zanetti *et al*, 1988; Elwood *et al*, 1990) and Oceania (Green *et al*, 1985; Holman *et al*, 1986), while similar studies on basal-cell carcinoma (BCC) and squamous-cell carcinoma (SCC) are sparser, with only three studies published in the 1990s of adequate size (Kricker *et al*, 1991; Gallagher *et al*, 1995a, 1995b; Rosso *et al*, 1996; Zanetti *et al*, 1996). The main difference between the three types of tumours involved their association with sun exposure: CMM is affected by intense and intermittent exposure to sun, while SCC by chronic sun exposure and BCC by both. However, these results have emerged from different methods of data collection: questionnaires, interviews, and clinical forms. Although questions and clinical examination focussed on similar aspects, the comparison of these results between the three types of skin tumours may have been affected by the different methods of data collection, classification and presentation, at some extent. Even within the same morphological class, comparison of different studies can be difficult, but, at least, in the case of CMM meta-analyses have helped to clarify relationships (Bliss *et al*, 1995; Elwood and Jopson, 1997; Whiteman *et al*, 2001; Gandini *et al*, 2005a, 2005b). The present study aimed at filling this gap with a multiple case–case–control design including CMM, BCC and SCC as case groups, using a control group, and testing differential risk by directly comparing the strength of association between pigmentary characteristics and sun exposure.

## MATERIALS AND METHODS

### Study design

This study was part of the Helios2 project mainly aimed at investigating the relationships between risk of skin tumours and amount and type of skin melanin (measured by its metabolites with HPLC method from hair sample), proposed as a more precise indicator of skin susceptibility (Zanetti *et al*, 2001). Cases and controls were recruited in 14 centres, mainly located in Europe: Turin, Biella, Florence, Naples, Ragusa (Italy); Granada, Murcia, Sevilla (Spain); Besançon, Montpellier (France); Lisboa (Portugal); Copenhagen (Denmark); Buxtehude (Germany); Buenos Aires (Argentina). The large number of recruitment centres, despite the relatively high frequency of these tumours, was to ensure a wide range of different melanic phenotypes, by sampling various populations. Each centre was assigned a minimum quota of cases for each cancer group and controls, proportional to its catchment area, in order to keep the recruitment time reasonably short and close to diagnosis, therefore assuring homogeneous conditions for risk assessment. Indeed, we interviewed all subjects from autumn 2001 to spring 2002, with a time lag from diagnosis ranging from 3 months to few days, and all centres reached their recruitment quota in less than 1 year (10 months).

We restricted our sample to Caucasian men from 20 to 75 years of age, since our principal aim was melanin determination in hair, and in a previous pilot phase (Zanetti *et al*, 2001) we found that a high proportion of women dyed their hair causing permanent alterations to the hair biochemical structure and therefore biasing biochemical assessment of melanin.

### Cases and controls

All cases presenting during the recruitment period to a clinic, hospital or outpatient department with suspected skin cancer and which met the eligibility criteria were identified and contacted by clinicians of each recruitment centre. Patients who agreed to be interviewed were then contacted by trained interviewers. Interviews could take place immediately during their hospital stays (40% CMM; 26% BCC; 35% SCC; and 64% controls), during a subsequent follow-up visit (43% CMM; 50% BCC; 40% SCC; and 23% controls), or elsewhere, usually at home (17% CMM; 24% BCC; 25% SCC; and 13% controls). We included all cases with a microscopically confirmed diagnosis of primary CMM, SCC or BCC. Prevalent cases were excluded: this information was gathered both directly during the interviews, and afterwards checking with local cancer registries (nine centres out of 14). All these cancer registries collected skin cancers routinely (Parkin *et al*, 2002), with the exception of Murcia and Montpellier which did not cover BCC. From pathology reports for melanoma we also recorded information on the site of lesion, histological morphology, and level of invasion (Clark's staging and Breslow's thickness).

Controls were chosen among patients admitted at same hospitals with non-dermatological diseases during the same period. Patients with orthopaedic injuries were excluded because of the possible association of their injuries with outdoor occupations or sport activities. Age of controls matched those of CMM and SCC (frequency matching). This study was approved by institutional review boards in each recruiting centre and all participants provided written informed consent.

### Exposure assessment

Interviewers were trained by courses held in different languages. The questionnaire gathered data on pigmentation and skin reaction to sun exposure, past experience of sunburn, lifelong exposure to sunlight during different recreational activities, occupational histories, cosmetic habits such as sunscreens and sun lamps, exposure to artificial UV sources for therapies, and dermatological history. Pigmentation was measured assessing hair and eye colour. We graded hair colour using a 11-level visual scale. Eye colour was assessed with a 10-level visual scale. Skin reaction to sun exposure was measured by ‘splitting’ the traditional Fitzpatrick's scale into two: tanning pattern and tendency to sunburn. We asked what type of tan (if any) and skin reaction the subject experienced when exposed to direct sunlight (without clouds) without protection (creams, clothes) for the first time since winter (or without any previous tanning, or parts of the body being at all tanned) and when exposed for an hour around midday during the summer. Past experience of painful and severe sunburns was assessed by asking questions on the number of lifelong sunburns, age at first sunburn and severity.

Interviewers counted the number of common naevi on the cases and controls forearms. A subjective indicator of global naevi count was also assessed with a visual chart presenting four body schemes with different densities of naevi approximately corresponding to none, less than 20, between 20 and 40 and more than 40. We asked the subjects which figures of the naevi chart best described their present situation of pigmented lesions. We used a similar visual chart to assess the appearance of freckles on the face when young, covering a range from none to heavy freckling in six increasing levels.

Interviewers asked questions on sun exposure using a structured questionnaire arranged by periods in life (before first employment, during active life and after retirement), places of residence for more than 6 months, and type of outdoor activity: work, holidays, sports or other outdoor recreational activities. Every time a subject referred to have spent more than 6 months working outdoor (for part-time activities, we considered the ones lasting at least 2 hours a day or 1 day a week), to have ever practised an outdoor sport or to have spent time outdoor during holidays, interviewers asked him for information on the number of years of activity, prevalent season of exposure (warmer and cooler months), hours of exposure (amount and distribution during daylight), and usual clothing during such activities. To help recall the type of clothing, questions were divided into body sections (trunk, upper and lower limbs). All these information were used for computing sun exposure indices based on cumulative time of exposure, as described in the following.

### Sun exposure indices

We estimated duration of exposure by summing up the number of hours spent in a lifetime in a particular outdoor activity. The different levels of sun irradiation were taken into account by assigning a weight proportional to the ratio of summer to winter irradiation in different places of exposure (on average, a ratio of 2). Sun exposure at a specific site was taken into account weighting total sun exposure by the proportion of body surface not protected by clothing. Weights were computed as proportions of exposed body surface to the whole body: head and neck representing about 9%, upper limbs 17%, lower limbs 35% and trunk 35% of the whole body surface. These indices were measured on continuous scales, but given the skewness of their distribution, we grouped original values by applying quartiles of cumulative distributions in exposed controls, that is, with exposure classes that divided exposed controls in almost equal numbers: 25% of total.

### Statistical analysis

We tested each case group separately by computing a set of separate comparisons with the control group, according to the approach proposed by Begg and Gray (1984). Factors were firstly analysed as category variables, and then, when measured on a multi-level, interval or continuous scale, we tested the presence of a linear trend, including them as continuous variables in a logistic model. In addition, linear trends of truly continuous variables such as hours of sun exposure were tested with variables in their original scale or after logarithmic transformation to normalise distributions.

Analysis, model building and testing of independent and differential effects was conducted as follows.

1. First, we analysed pigmentary characteristics, naevi and freckles by crude and model-adjusted odds ratios (OR_adj_). Adjustment was done introducing in a logistic regression model age, country of interview and all the variables from the first analysed set (i.e. host factors: pigmentary characteristics, naevi and freckles). In general, odds ratios were only slightly affected by adjustments by country of interview, showing measurement homogeneity across centres. Nevertheless, we left the ‘country’ (coded as: Spain, 3 centres; France, 2 centres; Italy, 5 centres; Germany/Denmark, 2 centres) as an adjustment parameter to take into account a potential residual confounding effect due to the possible differences in interview methods and cultural settings.
2. We then analysed the independent effect of host factors in separate binary logistic models, building a set of significant and independent factors for each case group.
3. Subsequently, we analysed sunburns and sun exposure, adjusting for age, country of interview and significant independent factors as emerged from the previous step on host factors.
4. The independent effect of sunburn and sun exposure was then tested as in step 2, with host factors included in the analysis.
5. Finally, the resulting variable set for all case groups was included in a multinomial logistic regression (MLR) (Dubin and Pasternack, 1986; Thomas *et al*, 1986) to test simultaneous effects and risk differences in each disease category.

## RESULTS

We collected information on 214 patients with CMM, 139 with SCC, 215 with BCC and 349 controls (Table 1). Participation was overall very good (84.8%), but better in controls (92.8%) than in cases (80.6%) and homogeneous across centres. Few cases died from other causes before being contacted. Most CMM were superficial spreading melanoma (59.3%) and few were of unknown type (11.7%). About a fifth (21%) were thin melanomas (below 0.5 mm), while 29% were above 2 mm. Body site of melanomas was mainly the trunk (50%), whereas BCC and SCC lesions were most frequently on the face and head (70% for both types of carcinomas). Recruited controls matched the conjoint distribution of CMM and SCC fairly closely up to the age of 40 years, where participation was higher, and with a difference of 6% in the 60–69 age group (Table 2).

### Host characteristics

Host characteristics are shown in Table 3 with frequencies, crude and adjusted odds ratios. Hair colour showed a highly significant association for all skin tumours, both considering model deviance when entering it as a multi-category variable (*P*-values: 0.0035 for CMM, 0.0027 for BCC and 0.0071 for SCC) or considering *P*-values for linear trend when entering it as an interval variable (Table 3). Pale eyes were also associated with a slight increase in risk. However, it was the green eye colour that showed the highest and significant risk, but only for CMM (OR_adj_: 3.2) and BCC (OR_adj_: 3.4). Tendency to tan showed the strongest and significant effect in SCC (OR_adj_:2.7 for people whose skin becomes red and does not tan), while in BCC the effect was less strong, although significant (OR_adj_: 1.5). Only a weak and not significant association was present in CMM. On the contrary, tendency to sunburn did not show an increase in risk for any of the skin tumours. Subjects with an elevated number of naevi had a high and consistent risk increase for CMM, when measured either with quantitative or qualitative naevi indicators which were highly correlated (polychoric correlation coefficient: 0.42). In Table 3 and in the following analyses we presented results for naevi measured with visual charts, as they showed the estimates with the narrowest confidence intervals, grouping visual chart categories in ‘some’ (a few to about 40 naevi) and ‘several’ (more than 40) *vs* none. Moles of over 10 mm were occasionally found, but the raised odds ratio was not significant. Freckles indicator showed a similar increased risk among CMM patients for all levels. For freckling we compared subjects with few to several freckles grouped together *vs* subjects with no freckles: risk of CMM was more than doubled and statistically significant (OR_adj_ 2.3, 95% CI 1.4–3.6), risk of BCC was only borderline significant (OR_adj_ 1.6, 95% CI 1.0–2.5), while no association was found for SCC.

### Sun exposure

Number of referred severe sunburns in a lifetime were grouped as 1–10 (sometimes) and more than 10 (often) and compared with subjects who never experienced sunburns. They showed a significant linear positive trend in BCC only, even after adjustment. Odds ratios for the other tumour types were not significant (Table 4). A linear increase in risk of SCC during outdoor work was present and statistically significant, and the most extreme exposure category, corresponding to more than 3878 weighted hours of exposure in a lifetime, showed a significant OR_adj_ of 2.2. For CMM, sun exposure during holidays at the beach showed a significant linear trend when introduced in models as a continuous variable. On the other hand, sun exposure during leisure time activities such as sports at the beach and outdoor sports in general showed only slightly above-unit but not significant odds ratio. Owing to this divergent direction of odds ratios, overall sun exposure did not show significant patterns for any of the tumour groups. Controlling by skin characteristics (separate models for disease group, selecting specific significant variables), did not substantially change odds ratios estimates in SCC and BCC, but noticeably weakened the effect of holidays at the beach in CMM models (Table 4).

In Table 5, for each type of tumour we showed the significant independent variables among those measuring host factors and sun exposures. For CMM they were fair hair, pale eyes, several naevi, and several freckles and frequent holidays at the beach. For SCC they were fair hair, no tanning and outdoor work. Finally, for BCC they were fair hair, pale eyes, frequent sunburns and holidays at the beach.

Although CMM models did not indicate an independent and significant risk increase for sunburns during childhood, sun exposure during outdoor activities in that period of life was associated with a significant risk increase (OR_adj_ 2.7, 95% CI 1.04–6.80; for more than 4700 weighted hours cumulated before adulthood). The same type of exposure during adult life did not have the same effect. In a similar way, BCC showed an increase in risk for sun exposure during holidays at the beach before adulthood (OR_adj_ 2.8, 95% CI 1.18–3.49) at even lower doses (for more than 3000 weighted hours), with a significant linear trend.

We also investigated the effect of variables interaction. Indeed, fair skin complexion is often associated to pale eyes and fair hair, difficult to tan, and tendency to burn when exposed to the sun. Also, most of the outdoor occupations are frequently associated with less opportunity for long vacations. However, we did not find evidence of statistically significant interactions, nor were odds ratios in logistic models substantially influenced by the introduction of interaction terms.

We also looked at the possibility of different risk patterns among CMM cases according to histology. The most relevant CMM group, with acceptable power for a statistical analysis (127 cases) was that of melanomas with superficial growth. Results indicated a slightly stronger effect of host characteristics (fair hair and pale eyes, freckles and naevi) but a decrease of sun exposure effect, after inclusion of all sun exposure indicators, including that of holidays at the beach during childhood (not significant any more). In the case of trunk melanomas (107 cases, of which 73 were superficial spreading melanoma), both the effects of host characteristics and sun exposure (holidays at the beach during childhood: OR_adj_ 5.2, 95% CI 1.88–14.31), increased significantly and substantially.

Finally, we simultaneously tested the differences in risk among tumour groups for all significant variables with a multinomial logistic model, presenting results in term of odds ratios of one cancer group (rows in Table 6) *vs* another (columns in Table 6) The findings are in accordance with the expected direction as shown from previous analyses: for subjects with an elevated number of naevi we found a 2.5 to a threefold risk increase in the CMM group, when compared to other skin cancers. On the other hand, SCC group was different in its higher association with tendency not to tan, with a 1.5 risk increase over the other two groups. Differences in other risk factors were in the same direction and at comparable levels, but without statistical significance. Exception was of sun exposure during holidays at the beach, with BCC showing a significant higher risk when compared to SCC.

## DISCUSSION

The three main types of skin tumours, melanomas, basal-cell and squamous-cell carcinomas, share the same risk factors. In brief, skin characteristics and sun exposure. However, similarities and differences have emerged from independent studies evaluating each tumour separately. Few studies comparing risk factors using the same measurement tools were available. Comparison between CMM and BCC, pooling data together from two previous case–control studies (Rosso *et al*, 1998), showed a differential risk increase (OR_adj_ 3.8, 95% CI 2.2–6.4) in melanoma patients who experienced sunburns during childhood, compared to patients with BCC (OR_adj_ 1.3, 95% CI 0.7–2.3); however, both tumours were sensitive to intense intermittent sun exposure (measured in hours of exposure at the beach during holidays), but with a different dose–response trend for CMM, where the risk increased only for very high lifelong cumulated doses.

A recent meta-analysis on melanoma and sun exposure (Gandini *et al*, 2005b) showed a significant combined OR of 1.61 (95% CI 1.31–1.99) for patterns of exposure to sun defined as ‘intermittent’, while ‘chronic’ exposure did not show any significant effect (OR 0.95, 95% CI 0.87–1.04). However, the definition of intermittent or occupational sun exposure, stemming from comparison of several different studies, could obviously yield only qualitative results without any reference to the amount of sun exposure.

In our study, activities implying a non-constant exposure to high doses of UV, such as those done during leisure time or holidays, showed an effect mainly before adulthood as in a previous study where we found a significant risk increase of 1.7 (95% CI 1.1–2.4) for a comparable exposure of more than 60 weeks of seaside holidays spent during childhood (Zanetti *et al*, 1992). A meta-analysis on melanoma and sun exposure during childhood showed similar results, although a summary odds ratio was proposed for ‘sunburn history’ only (OR 1.8, 95% CI 1.6–2.2) rather than for amount of sun exposure cumulated in the period (Whiteman *et al*, 2001). We did not find a risk increase for lifelong or childhood sunburns after controlling for other sun exposure variables. This is probably due to the more efficient measurement of sun exposure (Rosso *et al*, 2002) which has rendered the qualitative-only sunburn indicator inadequate: the strong link between sunburn and an intermittent pattern of sun exposure variables cancelled-out the sunburn indicator in the multivariate analysis.

The most relevant indicator of risk for CMM in the majority of the studies was the amount of common and atypical naevi and the presence of freckles: a systematic recent overview of results, found a pooled odds ratio of 1.5 (95% CI 1.36–1.59), even for a medium-low number of naevi (16–40), while for people with very high density of naevi (101–120) the pooled odds ratio was 6.9 (95% CI 4.63–10.25) (Gandini *et al*, 2005a).

The more sparse case–control studies of SCC and BCC produced comparable results. The Helios I project, from which the present study has originated, showed similar results, with a risk increase for outdoor work of about 1.6 (95% CI 0.93–2.75) in SCC, and in BCC a risk of about 1.5 (95% CI 1.18–1.83) for sun exposure during holidays at the beach (Rosso *et al*, 1996). Pigmentary characteristics in the Helios I study showed a stronger effect than presently reported, in particular, people with red hair with a risk of SCC 12 times higher than baseline (black/brown hair, who tan and do not burn) (Zanetti *et al*, 1996). In contrast, pale eye colour resulted in a higher risk for all skin tumours. However, we have to consider that eye colour is based on a different pigment (rodopsin), with a potentially different meaning and independent effect on the risk of skin tumours. A study in Canada (Alberta) of BCC and SCC showed comparable results (Gallagher *et al*, 1995a, 1995b), as did a study in Western Australia, but with slightly higher odds ratios for sun exposure than those found in this study (Kricker *et al*, 1991, 1995a, 1995b; English *et al*, 1998).

In conclusion, our direct case–case comparison to mitigate a possible bias in comparing results from different studies confirmed previous findings on the association between pale eyes, naevi and CMM, compared to other skin cancers, and the increased risk of BCC for intermittent sun exposure when compared to the risk of SCC.

## Figures and Tables

**Table 1:** Cases and controls by recruitment centres

	**Cases**						**Controls**	
	**CMM**		**SCC**		**BCC**			
**Centre**	**Interviewed**	**Refused, dead, untraceable**	**Interviewed**	**Refused, dead, untraceable**	**Interviewed**	**Refused, dead, untraceable**	**Interviewed**	**Refused, dead, untraceable**
Italian	69	14	36	17	65	6	109	8
Iberic and Latin American	87	13	73	12	94	43	156	12
French	29	2	16	4	21	7	43	7
German and Danish	29	1	14	7	35	11	41	0
								
Total	214	30	139	40	215	67	349	27

**Table 2:** Age distribution of cases and controls

**Age**	**CMM**	**SCC**	**CMM+SCC**	**BCC**	**Controls**	**Total**
20–29	15	1	16	0	17	33
30–39	39	1	40	5	40	85
40–49	37	9	46	32	56	134
50–59	60	26	86	72	99	257
60–69	51	67	118	83	95	296
70–75	12	35	47	23	42	112
						
Total	214	139	353	215	349	917

**Table 3:** Host factors and risk of skin tumours

	**Controls**	**CMM**			**SCC**			**BCC**		
	***N***	***N***	**Crude OR**	**Adj OR^a^**	***N***	**Crude OR**	**Adj OR^a^**	***N***	**Crude OR**	**Adj OR^a^**
*Hair colour*										
Dark	156	46	1	1	39	1	1	63	1	1
Brown	127	103	2.7	2.6	58	1.8	2.1	82	1.6	1.7
			(1.81–4.18)	(1.63–4.20)		(1.14–2.92)	(1.21–3.50)		(1.07–2.39)	(1.08–2.59)
Blonde	41	41	3.4	2.7	26	2.5	2.6	42	2.5	2.2
			(1.97–5.84)	(1.43–5.03)		(1.39–4.64)	(1.29–5.07)		(1.51–4.27)	(1.27–3.91)
Light blonde/Red	25	24	3.3	1.9	16	2.6	1.9	28	2.8	2.3
			(1.70–6.23)	(0.87–4.00)		(1.27–5.25)	(0.85–4.40)		(1.50–5.12)	(1.15–4.49)
*P*-linear trend			*P*<0.001	*P*<0.001		*P*=0.002	*P*=0.003		*P*<0.001	*P*<0.001
										
*Eye colour*										
Dark	110	44	1	1	28	1	1	37	1	1
Chesnut	96	57	1.5	1.2	33	1.3	1.2	48	1.5	1.3
			(0.92–2.40)	(0.70–2.17)		(0.76–2.40)	(0.62–2.16)		(0.89–2.47)	(0.75–2.18)
Green	42	50	3.0	3.2	26	2.4	1.9	56	4.0	3.4
			(1.74–5.10)	(1.69–6.04)		(1.28–4.62)	(0.90–3.95)		(2.29–6.85)	(1.92–6.22)
Blue/Grey	68	37	1.4	1.3	30	1.7	1.2	45	2.0	1.4
			(0.80–2.31)	(0.67–2.49)		(0.95–3.15)	(0.57–2.33)		(1.16–3.34)	(0.77–2.56)
Light blue	33	26	2.0	1.7	22	2.6	1.6	29	2.6	1.8
			(1.06–3.67)	(0.80–3.68)		(1.32–5.17)	(0.76–3.95)		(1.40–4.87)	(0.94–3.66)
*P*-linear trend			*P*=0.005	*P*<0.001		*P*=0.010	*P*=0.011		*P*<0.001	*P*<0.001
										
*Tendency to tan*										
Tan	178	89	1	1	40	1	1	90	1	1
No Tan	171	125	1.5	1.2	99	2.6	2.7	125	1.4	1.5
			(1.04–2.06)	(0.77–1.77)		(1.69–3.93)	(1.67–4.27)		(1.03–2.04)	(1.01–2.12)
										
*Tendency to sunburn*										
No sunburns	52	25	1	1	23	1	1	24	1	1
Sunburn/Blisters	297	189	1.3	0.8	116	0.9	0.6	191	1.4	1.0
			(0.79–2.21)	(0.46–1.48)		(0.52–1.51)	(0.31–1.06)		(0.83–2.34)	(0.59–1.78)
										
*Number of naevi (forearm count)*										
0	177	60	1	1	81	1	1	105	1	1
1–5	102	69	2.0	1.4	34	0.7	0.7	76	1.3	1.2
			(1.31–3.05)	(0.85–2.26)		(0.46–1.16)	(0.42–1.25)		(0.86–1.84)	(0.78–1.85)
										
6–10	32	38	3.5	1.9	14	1.0	1.1	16	0.8	0.8
			(2.01–6.10)	(0.98–3.63)		(0.48–1.89)	(0.49–2.50)		(0.44–1.61)	(0.37–1.61)
										
11+	38	47	3.6	1.5	10	0.6	0.4	18	0.8	0.7
			(2.17–6.13)	(0.72–2.82)		(0.27–1.21)	(0.16–1.13)		(0.43–1.47)	(0.35–1.47)
*P*-linear trend			*P*<0.001	*P*<0.001		*P*=0.176	*P*=0.530		*P*=0.697	*P*=0.754
										
*Number of moles>10 mm*										
No	327	193	1	1	126	1	1	195	1	1
Yes	22	21	1.6	0.9	13	1.5	2.0	20	1.5	1.6
			(0.87–3.02)	(0.41–1.99)		(0.75–3.14)	(0.83–4.68)		(0.81–2.86)	(0.79–3.32)
										
*Number of naevi (Chart)*										
No moles	140	30	1	1	70	1	1	78	1	1
Some	186	121	3.0	2.4	59	0.6	0.8	120	1.2	1.2
			(1.92–4.79)	(1.42–4.14)		(0.42–0.96)	(0.48–1.33)		(0.81–1.66)	(0.82–1.91)
Several	23	63	12.8	8.4	10	0.9	1.7	17	1.3	1.5
			(6.88–23.74)	(3.92–18.13)		(0.39–1.93)	(0.62–4.64)		(0.67–2.63)	(0.68–3.38)
*P*-linear trend			*P*<0.001	*P*<0.001		*P*=0.170	*P*=0.806		*P*=0.346	*P*=0.139
										
*Freckles*										
None	289	131	1	1	107	1	1	155	1	1
Some/Several	60	83	3.1	2.3	32	1.4	1.6	60	1.9	1.6
			(2.06–4.51)	(1.43–3.60)		(0.89–2.34)	(0.93–2.87)		(1.24–2.80)	(1.00–2.51)

a Adjusted by age and country of interview and all other variables in the table.

**Table 4:** Sunburn, sun exposure during outdoor activities and risk of skin tumours

	**Controls**	**CMM**			**SCC**			**BCC**		
	***N***	***N***	**Crude OR**	**Adj OR^a^**	***N***	**Crude OR**	**Adj OR^a^**	***N***	**Crude OR**	**Adj OR^a^**
*Lifelong sunburns*										
Never	180	87	1	1	71	1	1	91	1	1
Sometimes	121	87	1.5	1.1	50	1.0	1.3	79	1.3	1.3
			(1.02–2.82)	(0.67–1.65)		(0.68–1.61)	(0.79–2.20)		(0.88–1.89)	(0.87–2.07)
Often	48	40	1.7	0.8	18	0.9	0.8	45	1.8	1.5
			(1.05–2.82)	(0.44–1.50)		(0.52–1.74)	(0.40–1.62)		(1.15–2.99)	(0.87–2.62)
*P*-linear trend			*P*=0.008	*P*=0.032		*P*=0.909	*P*=0.335		*P*=0.011	*P*=0.04
										
*Sunburns in childhood*										
Never	308	173	1	1	128	1	1	176	1	1
Yes	41	41	1.8	0.9	11	0.6	0.7	39	1.7	1.3
			(1.11–2.85)	(0.53–1.65)		(0.32–1.29)	(0.33–1.58)		(1.03–2.68)	(0.77–2.28)
										
*Severe sunburns in childhood*										
Never/Rarely	308	174	1	1	117	1	1	173	1	1
Sometimes/Often	41	40	1.7	1.0	22	1.4	1.2	42	1.8	1.5
			(1.07–2.77)	(0.55–1.69)		(0.81–2.47)	(0.66–2.32)		(1.14–2.91)	(0.90–2.55)
										
*Outdoor work (No. of weighted hours in a lifetime)*										
Never	103	63	1	1	27	1	1	54	1	1
−320	60	46	1.2	1.1	17	1.1	1.3	44	1.4	1.4
			(0.75–2.02)	(0.70–1.87)		(0.54–2.11)	(0.62–2.77)		(0.83–2.89)	(0.83–2.51)
−1128	62	38	1.0	1.1	23	1.4	1.2	29	0.9	0.7
			(0.60–1.67)	(0.62–1.99)		(0.75–2.68)	(0.57–2.31)		(0.51–1.55)	(0.41–1.35)
−3878	62	36	0.9	1.1	29	1.8	1.8	44	1.3	1.4
			(0.57–1.59)	(0.63–2.06)		(0.97–3.29)	(0.92–3.54)		(0.81–2.25)	(0.80–2.40)
3878+	61	31	0.8	1.0	43	2.7	2.2	44	1.4	1.2
			(0.49–1.42)	(0.57–1.95)		(1.51–4.78)	(1.13–4.08)		(0.83–2.89)	(0.70–2.13)
*P*-linear trend			*P*=0.393	*P*=0.941		*P*=0.001	*P*=0.007		*P*=0.015	*P*=0.046
										
*Holidays (No. of weighted hours in a lifetime)*										
Never	60	26	1	1	43	1	1	41	1	1
−759	72	33	1.1	0.8	26	0.5	0.4	33	0.7	0.7
			(0.57–1.96)	(0.38–1.63)		(0.28–0.91)	(0.25–0.96)		(0.38–1.19)	(0.40–1.22)
−2253	72	54	1.7	1.2	21	0.4	0.5	41	0.8	1.0
			(0.97–3.09)	(0.60–2.40)		(0.22–0.76)	(0.25–1.07)		(0.48–1.45)	(0.54–1.82)
−4141	73	52	1.6	0.9	19	0.4	0.4	33	0.7	0.7
			(0.92–2.94)	(0.46–1.90)		(0.19–0.67)	(0.20–0.91)		(0.37–1.17)	(0.36–1.31)
4141+	72	49	1.6	1.1	30	0.6	0.6	67	1.4	1.3
			(0.87–2.82)	(0.52–2.23)		(0.33–1.04)	(0.29–1.21)		(0.81–2.29)	(0.72–2.39)
*P*-linear trend			*P*=0.050	*P*=0.077		*P*=0.033	*P*=0.050		*P*=0.171	*P*=0.171
										
*Holidays at the beach (No. of weighted hours in a lifetime)*										
Never	95	36	1	1	50	1	1	46	1	1
−648	64	39	1.6	1.3	26	0.8	0.9	46	1.5	1.5
			(0.92–2.79)	(0.67–2.42)		(0.44–1.36)	(0.49–1.77)		(0.88–2.49)	(0.84–2.60)
−1881	63	44	1.8	1.2	19	0.6	0.7	38	1.2	1.4
			(1.07–3.17)	(0.64–2.26)		(0.31–1.06)	(0.37–1.46)		(0.73–2.13)	(0.79–2.55)
−3592	63	42	1.8	1.0	19	0.6	0.7	28	0.9	1.0
			(1.02–3.04)	(0.53–1.99)		(0.31–1.06)	(0.35–1.47)		(0.52–1.62)	(0.51–1.80)
3592+	64	53	2.2	1.6	25	0.7	0.8	57	1.8	1.6
			(1.29–3.71)	(0.85–3.05)		(0.42–1.32)	(0.42–1.63)		(1.11–3.04)	(0.93–3.00)
*P*-linear trend			*P*=0.005	*P*=0.006		*P*=0.124	*P*=0.396		*P*=0.045	*P*=0.119
										
*Outdoor sports (No. of weighted hours in a lifetime)*										
Never	68	36	1	1	31	1	1	50	1	1
−351	70	47	1.3	1.0	34	1.1	1.3	32	0.6	0.7
			(0.73–2.19)	(0.53–2.00)		(0.59–1.92)	(0.67–2.61)		(0.36–1.08)	(0.38–1.29)
−1001	71	28	0.7	0.7	15	0.5	0.7	43	0.8	1.0
			(0.41–1.35)	(0.33–1.36)		(0.23–0.93)	(0.32–1.55)		(0.49–1.39)	(0.54–1.75)
−2783	69	58	1.6	1.5	31	1.0	1.3	43	0.8	0.9
			(0.93–2.71)	(0.79–2.98)		(0.54–1.79)	(0.65–2.61)		(0.50–1.44)	(0.50–1.67)
2783+	71	45	1.2	1.0	28	0.9	1.0	47	0.9	0.8
			(0.69–2.07)	(0.49–1.98)		(0.47–1.59)	(0.48–2.19)		(0.54–1.51)	(0.45–1.54)
*P*-linear trend			*P*=0.323	*P*=0.316		*P*=0.581	*P*=0.969		*P*=0.943	*P*=0.709
										
*Outdoor sports at the beach (No. of weighted hours in a lifetime)*										
Never	241	143	1	1	98	1	1	143	1	1
−132	27	15	0.9	0.7	9	0.8	1.1	8	0.5	0.5
			(0.48–1.82)	(0.35–1.56)		(0.37–1.81)	(0.48–2.47)		(0.22–1.13)	(0.19–1.16)
−664	27	17	1.1	1.3	9	0.8	0.7	26	1.6	1.4
			(0.56–2.01)	(0.62–2.67)		(0.37–1.81)	(0.29–1.60)		(0.91–2.89)	(0.78–2.67)
−2281	27	21	1.3	1.6	11	1.0	1.1	23	1.4	1.7
			(0.71–2.40)	(0.79–3.36)		(0.48–2.09)	(0.48–2.47)		(0.79–2.60)	(0.88–3.22)
2281+	27	18	1.1	1.2	12	1.1	1.2	15	0.9	1.0
			(0.60–2.11)	(0.58–2.42)		(0.53–2.24)	(0.55–2.82)		(0.48–1.82)	(0.49–2.06)
*P*-linear trend			*P*=0.458	*P*=0.407		*P*=0.979	*P*=0.803		*P*=0.360	*P*=0.443
										
*Overall sun exposure (No. of weighted hours in a lifetime)*										
0–2133	88	42	1	1	32	1	1	38	1	1
−4604	87	60	1.4	1.3	24	0.7	1.1	47	1.2	1.3
			(0.88–2.37)	(0.73–2.25)		(0.41–1.39)	(0.55–2.13)		(0.74–2.10)	(0.71–2.21)
−8788	88	54	1.3	1.2	30	0.9	1.0	61	1.6	1.6
			(0.78–2.12)	(0.73–2.25)		(0.52–1.67)	(0.53–1.96)		(0.97–2.65)	(0.93–2.85)
8788+	86	58	1.4	1.6	53	1.7	1.8	69	1.8	1.7
			(0.86–2.32)	(0.87–2.83)		(1.00–2.88)	(0.95–3.32)		(1.13–3.05)	(0.97–3.03)
*P*-linear trend			*P*=0.273	*P*=0.123		*P*=0.026	*P*=0.079		*P*=0.008	*P*=0.059

a Adjusted by age and country of interview and significant independent host factor variables.

**Table 5:** Odds ratio estimates from multiple logistic models with only significant and independent risk factors and terms for age by tumour type

	**CMM**	**SCC**	**BCC**
*Hair colour*			
Dark	1	1	1
Brown	2.3	2.0	1.6
	(1.42–3.56)	(1.18–3.25)	(1.07–2.52)
			
Blonde	2.3	2.4	2.3
	(1.27–4.26)	(1.26–4.64)	(1.32–3.94)
			
Light blonde/Red	1.8	2.1	2.6
	(0.85–3.75)	(0.97–4.67)	(1.39–5.02)
			
*Eyes colour*			
Dark	1	1	1
Green	2.6	NS	2.6
	(1.56–4.35)		(1.63–4.13)
			
*Tendency to tan*			
Tan	1	1	1
No Tan	NS	2.6	NS
		(1.62–4.04)	
			
*Number of naevi (Chart)*			
No moles	1	1	1
Some	2.3	NS	NS
	(1.46–3.80)		
Several	8.7	NS	NS
	(4.52–16.71)		
			
*Freckles*			
None	1	1	1
Some/Several	2.1	NS	NS
	(1.37–3.35)		
			
*Lifelong sunburns*			
Never	1	1	1
Sometimes	NS	NS	1.4
			(1.01–2.42)
Often	NS	NS	1.7
			(1.01–2.82)
			
*Holidays at the beach (No. of weighted hours in a lifetime)*			
<7000	1	1	1
7000+	2.6	NS	2.1
	(1.14–5.71)		(1.09–3.95)
			
*Outdoor work (No. of weighted hours in a lifetime)*			
<6000	1	1	1
6000+	NS	2.2	NS
		(1.24–4.06)	

NS, not included in the multiple logistic model since not significant.

**Table 6:** Pairwise comparisons of selected risk factor effects between skin cancer groups

	**Odds ratio differences (row vs column)**		
	**CMM**	**SCC**	**BCC**
*Hair colour*			
CMM	—	1.031	1.014
SCC		—	0.984
BCC			—
			
*Eyes colour*			
CMM	—	1.576	0.880
SCC		—	0.559*
BCC			—
			
*Tendency to tan*			
CMM	—	0.640*	0.962
SCC		—	1.504*
BCC			—
			
*Naevi*			
CMM	—	3.037*	2.677*
SCC		—	0.806
BCC			—
			
*Freckles*			
CMM	—	1.066	1.016
SCC		—	0.954
BCC			—
			
*Lifelong sunburns*			
CMM	—	1.051	0.952
SCC		—	0.861
BCC			—
			
*Holidays at the beach (No. of weighted hours in a lifetime)*			
CMM	—	1.376	0.924
SCC		—	0.671*
BCC			—
			
*Holidays at the beach during childhood (No. of weighted hours in a lifetime)*			
CMM	—	4.110	1.285
SCC		—	0.313
BCC			—
			
*Outdoor work (No. of weighted hours in a lifetime)*			
CMM	—	0.857	0.946
SCC		—	1.103
BCC			—

* *P*<0.05.

## Appendix A. The Helios Working Group

*Coordinating Centre:* Piedmont Cancer Registry, CPO, Torino (R Zanetti, Project Leader; S Rosso, C Sacerdote).

*Recruitment Centres*

*Italy*: Dermatology Department, University of Turin (M Pippione and P Broganelli); ‘Infermi’ Hospital, Biella (C Barbera, R Manzoni and P Pella); Regional Agency for Public Health of Tuscany, Florence (E Buiatti and D Balzi); Department of Dermatology, University of Florence (P Carli and A Chiarugi); Dermatology Department, ‘Federico II’ University, Neaples (P Santoianni, G Fabbrocini and E Barberio); Italian League Against Cancer – Ragusa Section (L Gafà and C Lauria). *Spain:* Murcia Cancer Registry, Murcia (C Navarro, MD Chirlaque and MJ Tormo-Diaz); Granada Cancer Registry, Granada (C Martinez and M J Sanchez); Faculty of Medicine, University of Sevilla (A Nieto, L Abdel-Kader, F Camacho and JR Armas). *France:* Doubs Cancer Registry, Faculty of Medicine and Pharmacy University of Besançon (M Mercier and P Louvat); Department of Dermatology, University of Besançon (F Aubin); Hérault Cancer Registry, Montpellier (H Sancho-Garnier and B Trétarre). *Portugal:* Southern Portugal Regional Oncological Institute, Lisboa (A Miranda). *Argentina:* Roffo Institute of Oncology and Pirovano Hospital, Buenos Aires (DI Loria, N Zengarini and J Garau). *Germany:* Dermatological Hospital, Krankenhaus Buxtehude (EW Breitbart and R Greinert). *Denmark:* Dermatology Clinic, Hillerød (A Østerlind).

*Laboratory:* Institute for macromolecules (ISMAC), National Research Council (CNR), Biella (M Zoccola, R Mossotti and R Innocenti).
